# Multi Frequency Controllable In-Band Suppressions in a Broad Bandwidth Microstrip Filter Design for 5G Wi-Fi and Satellite Communication Systems Utilizing a Quad-Mode Stub-Loaded Resonator

**DOI:** 10.3390/mi14040866

**Published:** 2023-04-17

**Authors:** Guoqiang Zhang, Abdul Basit, Muhammad Irshad Khan, Amil Daraz, Najmus Saqib, Farid Zubir

**Affiliations:** 1 School of Information Science and Engineering, NingboTech University, Ningbo 315100, China; guoqiang_zhang@nbt.edu.cn (G.Z.); amil.daraz@nbt.edu.cn (A.D.); 2 College of Information Science and Electronic Engineering, Zhejiang University, Hangzhou 310027, China; 3 College of Electronics and Information Engineering, Nanjing University of Aeronautics and Astronautics (NUAA), Nanjing 210000, China; irshadnawab@nuaa.edu.cn; 4 Department of Electrical Engineering, University of Engineering and Technology, Peshawar 25000, Pakistan; najmussaqib@uetpeshawar.edu.pk; 5 Wireless Communication Centre, Faculty of Electrical Engineering, Universiti Teknologi Malaysia, Johor Bahru 81310, Malaysia; faridzubir@utm.my

**Keywords:** uniform transmission line, super wideband, bandpass filter, wireless communication, triple notched bands, T-shaped shorted stub-loaded resonators, low insertion loss

## Abstract

The key elements used for receiving and processing signals in communication systems are the bandpass filters. Initially, a common operating mechanism was applied for the design of broadband filters, i.e., by cascading low-pass filters or high-pass filters using multiple line resonators with length quarter-half- or full-wavelength with central frequency, but using these approaches, the design topology becomes expensive and complex. The above mechanisms can be possibly overcome using a planar microstrip transmission line structure due to its simple design fabrication procedure and low cost. So, pointing out the above problems in bandpass filters such as low-cost, low insertion loss, and good out-of-band performance, this article presents a broadband filter with multifrequency suppression capability at 4.9 GHz, 8.3 GHz, and 11.5 GHz using a T-shaped shorted stub-loaded resonator with a central square ring coupled to the basic broadband filter. Initially, the C-shaped resonator is utilized for the formation of a stopband at 8.3 GHz for a satellite communication system, and then a shorted square ring resonator is added to the existing C-shaped structure for the realization of two more stopbands at 4.9 GHz and 11.5 GHz for 5G (WLAN 802.11j) communication, respectively. The overall circuit area covered with the proposed filter is 0.52 λg × 0.32 λg (λg is the wavelength of the feed lines at frequency 4.9 GHz). All the loaded stubs are folded in order to save the circuit area, which is an important requirement of next-generation wireless communication systems. The proposed filter has been analyzed using a well-known transmission line theory, even–odd-mode, and simulated with the 3D software HFSS. After the parametric analysis, some attractive features were obtained, i.e., compact structure, simple planar topology, low insertion losses of 0.4 dB over the entire band, good return loss greater than 10 dB, and independently controlled mutli stopbands, which make the proposed design unique and can be used in various wireless communication system applications. Finally, a Rogers RO-4350 substrate is selected for the fabrication of the prototype using an LPKF S63 ProtoLaser machine and then measured using a ZNB20 vector network analyzer for matching the simulated and measured results. After testing the prototype, a good agreement was found between the results.

## 1. Introduction

Recently, low cost, enhanced out-of-band rejection, and low losses are highly recommended for the design of wide passband filters as these play an important role in the integration with other circuits/antennas, etc., to enhance the performance of the radio communication systems studied in [1,2,3]. Only a few procedures have been used by microwave researchers for the design of wideband filters in recent decades [4,5,6,7,8,9,10,11,12,13,14]. For example, the authors of [4,5,6,7] utilized different topologies such as a defective ground structure (DGS) and funnel-type asymmetric resonator for a wideband bandpass filter (BPF) with an upper wide stopband response. Another λ/2 circular microstrip quadruple/quintuple-mode resonator with parallel-coupled microstrip lines has been utilized by [8] to develop a wideband filter with fractional bandwidth (FBW) of 60% and 62%, but the proposed structures have high insertion loss (IL). The authors of [9] achieved a good FBW of about 177% in a wideband filter range from 0.29 GHz to 4.82 GHz using a grating array and interdigital strip structure. The drawback seen in this design is the complex geometry, although it has a good passband response. A triple-notched wideband BPF (bandpass filter) ranging from 2.2 GHz to 7.6 GHz is designed and fabricated with enhanced upper-frequency band suppression using interdigital lines and DGS on the back of the Rogers 4350 substrate. Four transmission zeros (TZs) were achieved outside the passband of the filter to enhance the upper stopband suppression up to 32 GHz. However, the presented filter utilizes a very complex geometry that can lead to a precise fabrication measurement [10]. Another staircase resonator was used by the authors of [11] to fabricate a wideband filter with an FBW of 62.3%. Recently, a wideband filter response with an FBW of about 132% was achieved using an H-type sandwich slot-line structure in [12]. The selectivity of the filter was greatly increased by introducing a source-to-load coupling, but this increased the IL in the passband. The structure was fabricated on Rogers 5880 PCB with an overall covered area of about 32 × 15 mm. A tapered transmission line resonator (TTR) was utilized in [13] for the implementation of a high selectivity UWB (ultra-wideband) response with an FBW of 112%, IL of 1 dB, and return loss better than 17 dB. It was noticed that the TTR reduced the circuit size and that a good controllable BW was achieved, but the author used a very complex geometry to build the UWB filter, and improvement is still required in the passband. Another wideband filter was designed by the authors of [14] by cascading a low-pass filter and a high-pass filter. The structure has a good IL of 0.4 dB and good return loss, but the FBW is low, i.e., 107%. The advantage of this prototype is to suppress the unwanted frequencies in the upper stopband up to 20 GHz.

There is still a demand for microwave filters to improve the above requirements using a simple design methodology that allows easy implementation of the wideband filters. In this regard, a filter with triple-notched bands has been explored in this research work using a T-shaped shorted stub-loaded resonator with a central square ring coupled to the initial SUWB-BPF (super ultra-wideband bandpass filter) for the rejection of radio frequency bands at 4.9 GHz, 8.3 GHz, and 11.5 GHz. The basic ultra-wideband filter is constructed using a uniform transmission line (UTL) loaded with three folded λ/4 short-circuited shunt stubs with pads; out of this, one stub is placed in the middle section of one side of the UTL, and the other two are placed at the symmetrical position on the opposite side of the UTL. The proposed filter covered an area of 22.5 mm × 12 mm (excluding feed lines). Finally, the prototype was fabricated on a low-cost PCB and tested with VNA, and the results obtained were closely matched with a simulation. A 3D electromagnetic (EM) simulation software Ansysis HFSS version-15 was used for simulations in this study [15].

**Figure 1:**
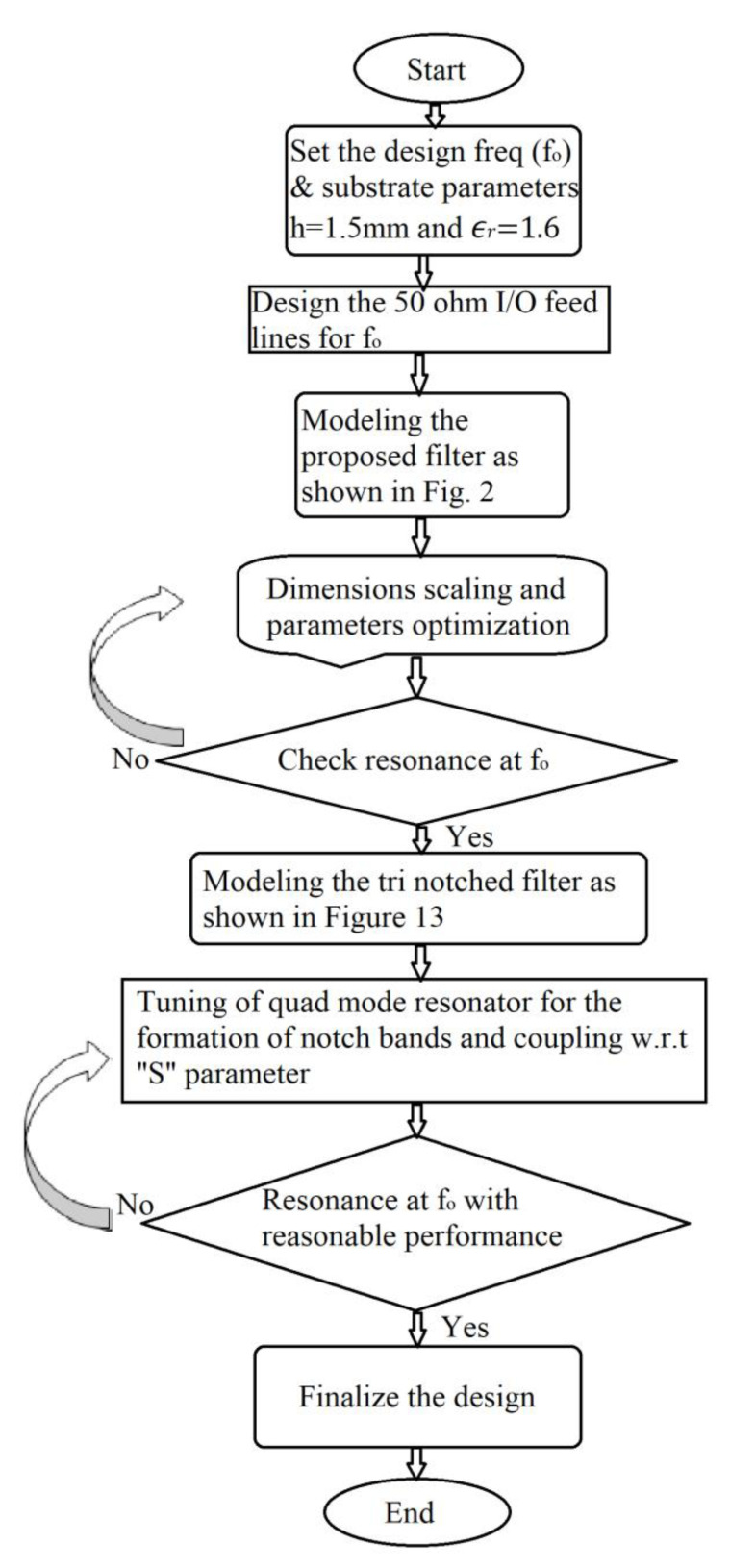
Design flow chart.

This research work was completed in the following way: Section 2 describes the complete analysis of the UTL, and Section 3 describes how the SUWB-BPF with in-band controllable frequency bands was designed. Section 4 shows the proposed topology and how the resonance frequencies are theoretically found and experimentally verified. Section 5 validates the measured and experimental work, while Section 6 concludes the research work. Moreover, a design flow chart showing how this research work was completed is shown in Figure 1.

## 2. Design and Analysis of the SUWB-BPF

**Figure 2:**
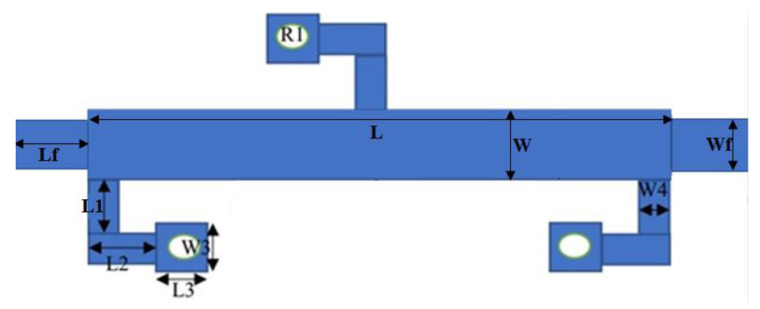
Proposed SUWB-BPF architecture.

**Figure 3:**
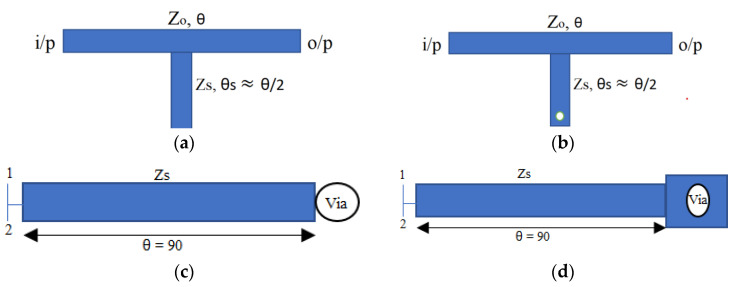
Stubs configurations: (**a**) open stub connected to UTL, (**b**) short-circuited stub connected to UTL, (**c**) conventional stub, and (**d**) the proposed shunt stub.

The architecture of the basic SUWB-BPF is shown in Figure 2. It is constructed using a UTL loaded with three folded λ/4 short-circuited shunt stubs with pads. Out of this, one stub is placed in the middle section of one side of the UTL, and the other two are placed at the symmetrical position on the opposite side of the UTL. The analysis of the uniform impedance resonator is carried out by considering a half-wavelength resonator connected with open- or short-circuited stubs in the middle, as shown in Figure 3a,b. Assuming no losses, the network resonance condition can be determined with the transfer matrix [*ABCD*]. The expression of the input admittance is [16,17]:

(1) $$Y_{in}=\frac{1}{Z_{11}}=\frac{C}{A}=0$$

where *Z*_11_ is the input impedance of the resonator. The resonance Equation (1) is identical to the requirement for resonators when the zeros of elements *C* and *A* in the transfer matrix do not coincide and *A* does not contain poles that are different from those in *C*.

(2) C=0

The output admittance (*Y_out_*) also becomes zero when the condition in Equation (2) is satisfied. Therefore,

(3) $$Y_{out}=\frac{1}{Z_{22}}=\frac{C}{D}=0$$

The generalized transfer matrix [*ABCD*] in the left portion of Figure 3a,b in terms of transfer matrices of the elements *A*′, *B*′, *C*′*, D*′ is [18]:

(4) $$\left(\begin{array}{cc} A & B\\ C & D \end{array}\right)=\left(\begin{array}{cc} 1+2B^{\prime }C^{\prime } & 2A^{\prime }B^{\prime }\\ 2C^{\prime }D^{\prime } & 1+2B^{\prime }C^{\prime } \end{array}\right)$$

Equation (4) represents the resonance condition of Equation (2) that satisfies the following two conditions:

(5) $$C^{\prime }=0$$

(6) $$D^{\prime }=0$$

The above two conditions are simpler to perform in analysis than the traditionally used *Y_in_* = 0. Using the resonators depicted in Figure 3a,b, let us construct the resonance equations. At the point where the stub connects to the TL (transmission line) segment, we utilize the input admittance *Ys* of the stub and the transfer matrix [*abcd*] of the left half of the TL segment. The matrix [*A*′*B*′*C*′*D*′] is clearly seen as the result of two matrices [19,20]:

(7) $$\left(\begin{array}{cc} A^{\prime } & B^{\prime }\\ C^{\prime } & D^{\prime } \end{array}\right)=\left(\begin{array}{cc} a & b\\ c & d \end{array}\right)\left(\begin{array}{cc} 1 & 0\\ \frac{Y_{s}}{2} & 1 \end{array}\right)=\left(\begin{array}{cc} a+\frac{bY_{s}}{2} & b\\ c+d\frac{Y_{s}}{2} & d \end{array}\right)$$

The above chain equation follows the expression (3), i.e.,:

$$d=0\;Y_{s}=\frac{-2c}{d}$$

The matrix [*abcd*] may generally represent a portion of a non-uniform TL. When a segment with a uniform TL is used, its electrical length and characteristic impedance *Z*_0_ are:

(8) $$\left(\begin{array}{cc} a & b\\ c & d \end{array}\right)=\left(\begin{array}{cc} \cos\frac{\theta }{2} & jZ_{o}\sin\frac{\theta }{2}\\ Z_{o}{}^{-1}j\sin\frac{\theta }{2} & \cos\frac{\theta }{2} \end{array}\right)$$

Applying resonance conditions on the above equation:

(9) $$\cos\frac{\theta }{2}$$

(10) $$Y_{s}=-j2Z_{o}{}^{-1}\tan\frac{\theta }{2}$$

The resonant electrical length of Equation (9) is:

$$\theta_{n}=\pi ,3\pi ,5\pi$$

**Figure 4:**
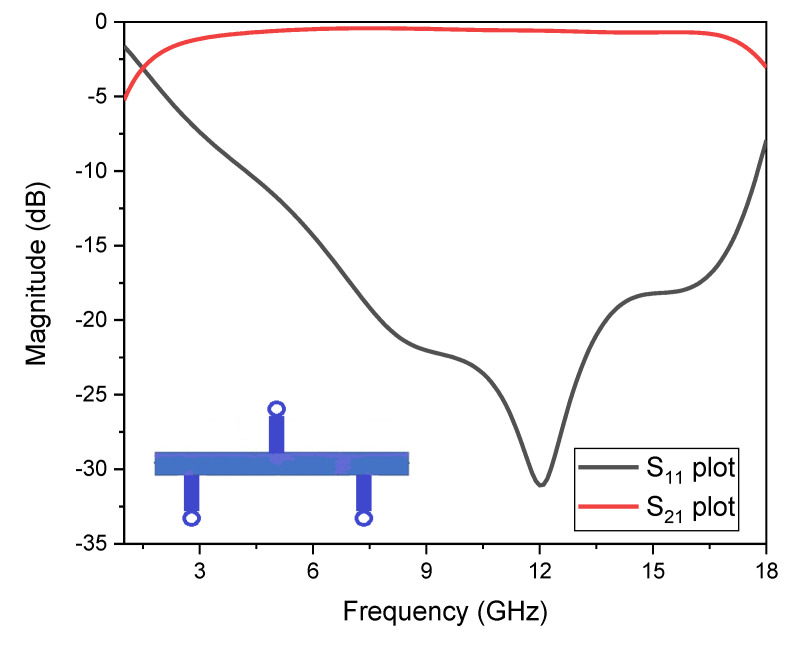
S_11_ vs. S_21_ frequency plot by loading conventional stub.

The transcendental Equation (10) determines the other component of the resonant electrical lengths, which is dependent on the stub parameters. The same analysis can be used for the short-circuited stub of length *θs* << *θ*/2 in Figure 3b, but only Equation (10) takes the below form, while Equation (9) is still applicable:

$$-Z_{s}{}^{-1}\cot\theta_{s}=-2Z_{o}{}^{-1}\tan\frac{\theta }{2}$$

where *θ* = βL shows the electrical length of the stubs with physical length L and propagation constant β. Now, the configuration in Figure 3a,b is replaced with the stubs proposed in [21], as shown in Figure 3c,d. Initially, conventional stubs, as shown in Figure 3c, were used at the center of the UTL. Due to this arrangement, a wideband filtering response with poor sharpness and one TP (transmission pole) at 12 GHz was observed, as shown in Figure 4. The corresponding transmission matrix of the conventional stub in Figure 3c is given below [21]:

(11) $$[A]=\left(\begin{array}{cc} 1 & 0\\ j\frac{1}{Zs}\tan\theta & 1 \end{array}\right)$$

In order to achieve a higher degree of freedom in the design, the conventional stub is replaced with a new folded shunt stub with pads, as shown in Figure 3d, and two more of the same stubs are placed symmetrically on the opposite side of the UTL at a distance equal to λ/2, and thus, a modified topology is obtained, as shown in Figure 2, respectively. The corresponding transmission matrix of the proposed stub in Figure 3d is given below:

(12) $$[A]=\left(\begin{array}{cc} 1 & 0\\ -j\frac{1}{Zs}\cot\theta & 1 \end{array}\right)$$

**Figure 5:**
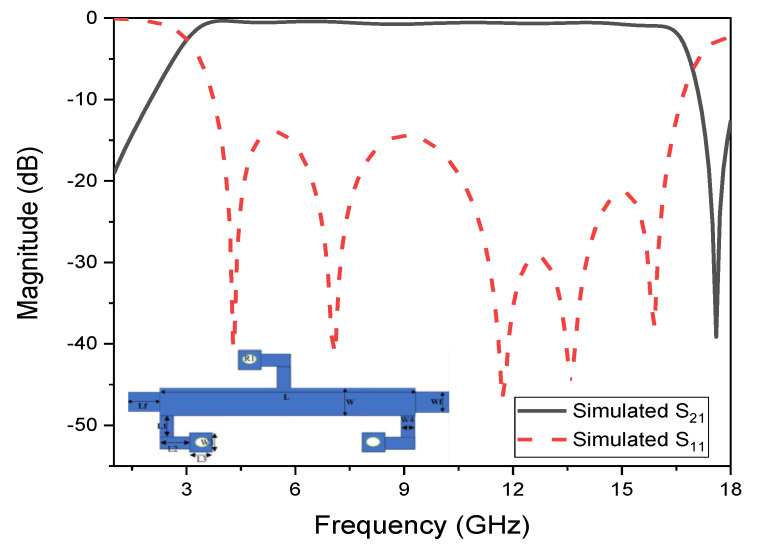
Simulated frequency plots of the SUWB-BPF with proposed rectangular pads.

Due to this arrangement of the stubs, the performance of the filter is greatly improved, as shown in Figure 5, in terms of its wide flat passband, excellent IL, and good sharp rejection level with two TZs at the lower and upper stopband frequency, and five TPs appear within the passband at different frequencies, respectively. The reflection zeros will appear at (2n − 1)f_o_, while the transmission zeros appear at 2nf_o_. 

## 3. Operational Principle of the SUWB-BPF with In-Band Frequency Suppression

**Figure 6:**
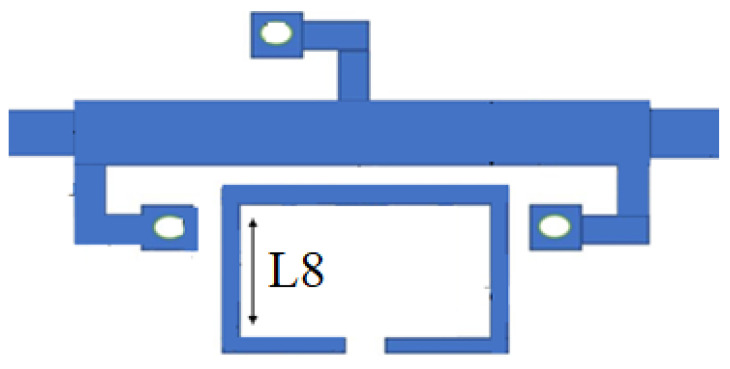
The proposed C-shaped resonator topology for the 8.3 GHz stopband.

**Figure 7:**
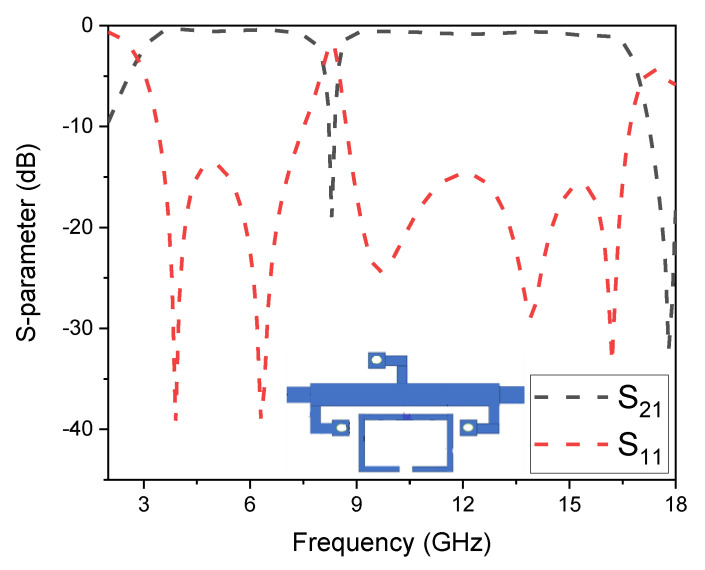
Response of the proposed C-shaped resonator at 8.3 GHz.

**Figure 8:**
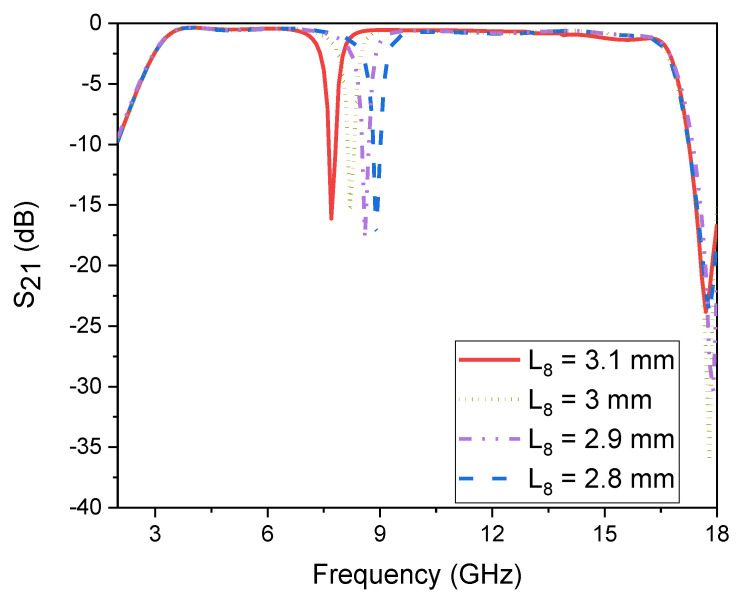
Control of the C-shaped resonator for the 8.3 GHz stopband.

**Figure 9:**
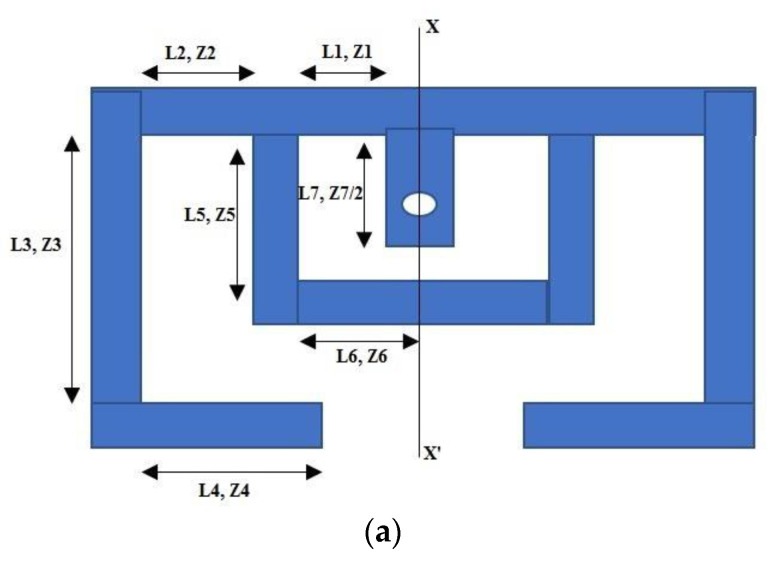

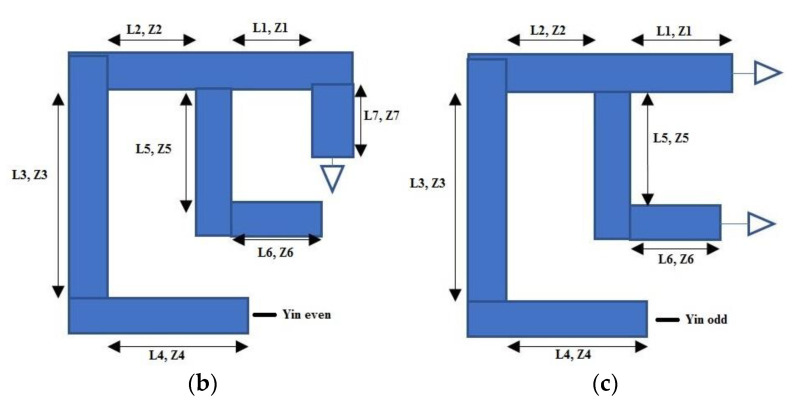
(**a**) The presented geometry of the quad-mode stub-loaded resonator. (**b**) Even-mode excitation circuit. (**c**) Odd-mode excitation circuit.

A novel T-shaped shorted stub-loaded resonator with a central square ring, also known as a quad-mode stub-loaded resonator, was utilized for the realization of in-band frequency suppression in the SUWB-BPF. The operating principle for the formation of notch bands was completed in two steps. Initially, the folded C-shaped resonator, as shown in Figure 6, was coupled to the basic wideband filter, and after optimizing the length of the proposed resonator, a single stop band at 8.3 GHz was obtained, as depicted in Figure 7 with its control shown in Figure 8. In the second step, a centrally shorted square ring was loaded to the C-shaped structure for the realization of two more stopbands at 4.9 GHz and 11.5 GHz. In this way, a combination of the C-shaped resonator and square ring resonator was obtained, which is also called a T-shaped shorted stub-loaded resonator with a central square ring or a quad-mode stub-loaded resonator. The name quad-mode stub-loaded resonator is given by the authors of this work as it generates four resonant modes, which will be explained in the section below. The complete configuration of the proposed resonator for the triband responses is shown in Figure 9a, which is composed of a ring resonator, a common half wavelength (λ/2) uniform-impedance resonator (UIR), and a short stub. It can be seen that the proposed resonator is symmetrical with respect to the central vertical plane XX’; therefore, a well-known classical method called the even–odd-mode is performed to characterize the resonance behavior of the proposed architecture discussed in [22,23]. This method will simplify the mathematical calculation by dividing the resonator into two sections along the XX’ plane; one section will behave as a magnetic wall (M.W.), called even mode excitation, while the other one behaves as an electric wall (E.W.), called odd mode excitation, as shown in Figure 9b,c, respectively. According to the conventional theory of the even–odd-mode analysis, the resonator on the symmetrical plane XX’ is shorted under odd mode excitation and open-circuited under even mode excitation, but in this topology, the plane XX’ for the even mode excitation is shorted with one end due to it already being present in the topology, as shown in Figure 9a.

**Figure 10:**
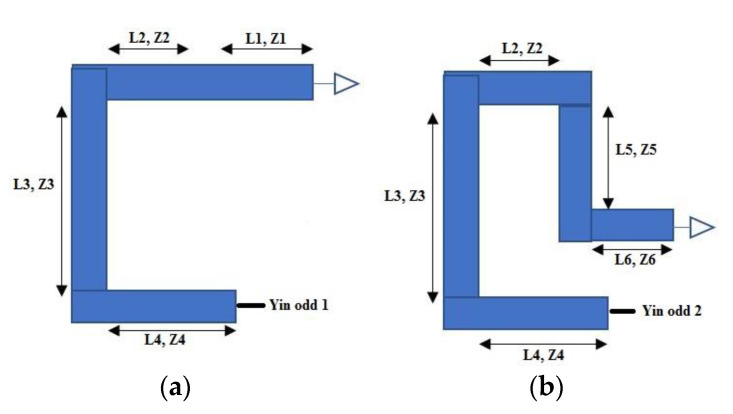
(**a**) First odd-mode excitation circuit. (**b**) Second odd-mode excitation circuit.

The odd-mode equivalent circuit in Figure 9c consists of two quarter wavelength paths with one end grounded, as shown in Figure 10a,b. Thus, two resonance frequencies *f*_*odd*1_ and *f*_*odd*2_ can be realized by setting the resonance condition, i.e., *Y*_*in odd*1_ = 0 and *Y*_*in-odd*2_ = 0. According to the basic microwave network theory, the input admittance of the odd modes can be calculated using the mathematical equation below [24]:

(13) $$Y_{in}=Y_{0}\frac{Y_{L}+jY_{0}\tan\theta }{Y_{0}+jY_{L}\tan\theta }$$

(14) $$Y_{in}=Y_{0}\frac{Y_{L}+jY_{0}\tan(L2\pi /\lambda_{g})}{Y_{0}+jY_{L}\tan(L2\pi /\lambda_{g})}$$

where $\lambda_{g}=\frac{C}{f\sqrt{\varepsilon_{eff}}}$ and

$$\varepsilon_{eff}=\frac{1+\varepsilon_{r}}{2}+\frac{\varepsilon_{r}-1}{2}\times \frac{1}{\sqrt{1+12\frac{h}{w}}}$$

(15) $$Y_{in,odd1}=\frac{Y_{1}}{j\tan(\theta_{1}+\theta_{2}+\theta_{3}+\theta_{4})}$$

(16) $$f_{odd}{}_{1}=\frac{c}{4(L_{1}+L_{2}+L_{3}+L_{4})\sqrt{\varepsilon_{eff}}}$$

(17) $$Y_{in,odd2}=-jY_{1}[\frac{Y_{2}\tan(\theta_{5}+\theta_{6}-\pi /2)+Y_{in-odd1}}{Y_{1}+j[(Y_{2}\tan(\theta_{5}+\theta_{6}-\pi /2)\tan(\theta_{1}+\theta_{2}+\theta_{3}+\theta_{4})]}]$$

(18) $$f_{odd2}=\frac{(2n+1)c}{4(L_{2}+L_{3}+L_{4}+L_{5}+L_{6})\sqrt{\varepsilon_{eff}}}$$

**Figure 11:**
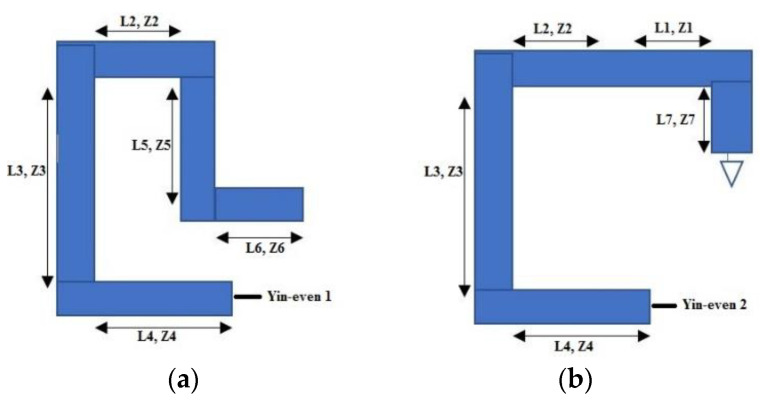
(**a**) First even-mode excitation circuit. (**b**) Second even-mode excitation circuit.

For even-mode excitation, the circuit in Figure 9b also contains two paths: a λ/2 resonator with one end open constitutes path-I, as shown in Figure 11a, while a λ/4 resonator with one end grounded constitutes path-II, as illustrated in Figure 11b, respectively.

Using the same basic microwave network theory discussed in the odd-mode case, two resonance frequencies *f_even_*_1_ and *f_even_*_2_ will be excited by setting the resonance condition, i.e., *Y_in even_*_2_ = 0 and *Y_in even_*_2_ = 0.

(19) $$f_{even1}=\frac{(2n-1)c}{2(L_{2}+L_{3}+L_{4}+L_{5}+L_{6})\sqrt{\varepsilon_{eff}}}$$

(20) $$f_{even2}=\frac{(2n-1)c}{4(L_{1}+L_{2}+L_{3}+L_{4}+L_{7})\sqrt{\varepsilon_{eff}}}$$

To reduce the complexity of the mathematical calculation, assume *Z*_1_ = *Z*_2_ = *Z*_3_ = *Z*_4_ = *Z*_5_. It can be seen that *f*_e1_ = 2*f*_02_, so a total of three resonance frequencies are obtained. The stopbands can be controlled with the parameters that appeared in the denominator of Equations (16) and (18)–(20), respectively, which will be discussed in the next section.

## 4. Proposed Filter Architecture and Determination of Stopband Frequencies

**Figure 12:**
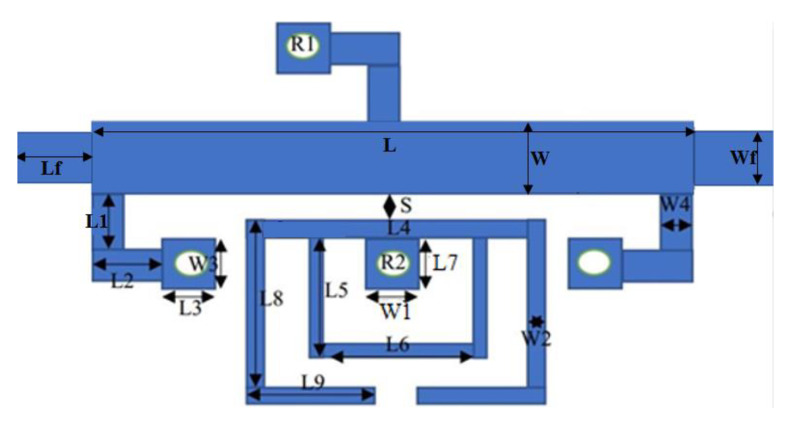
The presented architecture of the SUWB-BPF with a quad-mode stub-loaded resonator.

This section describes the specification of the proposed filter architecture and the logic behind the formation of stopband frequencies using the mathematical model analyzed in Section 3. The filter is made up of a UTL loaded with three folded λ/4 short-circuited rectangular pads. Out of these, one stub placed in the middle section of one side of the UTL, and the other two are placed at the symmetrical position on the opposite side of the UTL. The stubs have been placed at a distance of a half wavelength from each other to achieve five TPs in the passband. The layout of the proposed filter is shown in Figure 12 with optimized dimensions in millimeters (mm) displayed in Table 1. The stub arrangement made the proposed filter compact with a covered area of 0.52 *λ*_g_ × 0.32 *λ*_g_. It has been fabricated on Roger 4350 substrate material, with specifications listed in Table 1, and analyzed using a ZNB20 vector network analyzer. For the rejection of radio frequency bands at 4.9 GHz, 8.3 GHz, and 11.5 GHz, the stopband characteristics have been attained utilizing a quad-mode stub-loaded resonator coupled to the basic filter structure in Figure 2. All the stopbands can be independently controlled with the help of the lengths mentioned in the respective equations using the corresponding mathematical models listed in Table 2. Therefore, the resonance frequencies in the proposed resonator are controlled independently and mathematically verified, simulated, and then fabricated using a low-loss substrate material.

## 5. Measured and Experimental Results

As discussed, the SUWB BPF is constructed using a UTL loaded with three folded λ/4 short-circuited shunt stubs, which are placed symmetrically at a distance equal to λ/2 from each other. Three stubs were placed symmetrically on either side of the UTL in order to achieve a high degree of freedom in the design. Due to this arrangement of the stubs, the performance of the filter was greatly improved in terms of its wide flat passband with an FBW of 141.1% or absolute bandwidth of 13.9 GHz, excellent IL of less than 0.4 dB, and good sharp rejection level at lower and upper frequency, and five TPs appear within the passband at 4.5 GHz, 7.3 GHz, 11.7 GHz, 13.8 GHz, and 16.4 GHz, respectively. 

For the generation of notches in the initial wideband filter, a symmetrical quad-mode stub-loaded resonator was used. In the first step, a C-shaped resonator was utilized for the second band formation at 8.3 GHz, and then a centrally shorted square ring resonator was added to the C-shaped resonator for the realization of the first and third notch bands at 4.9 GHz and 11.5 GHz, respectively. The final resonator topology obtained was called a quad-mode stub-loaded resonator. Due to its symmetrical nature along XX’, a classical method called even–odd-mode was applied to study the resonance behavior of the resonator. After a detailed mathematical calculation, four resonance frequencies were observed, out of which, two were generated with the help of odd mode analysis, and two were observed with the help of even mode analysis. It was assumed that f_e1_ = 2f_02_ by considering all impedances to be equal. So, a total of three resonance frequencies were obtained, which lead to the construct of a triple-notch wideband BPF. 

**Figure 13:**
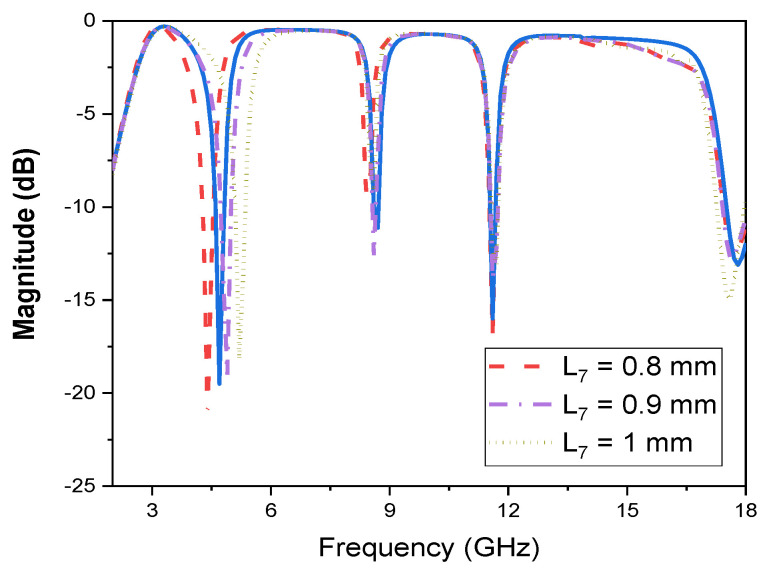
Validation of first band control w.r.t.

The first notch at 4.9 GHz was realized due to the second fundamental even mode of the resonator and by varying the parameter *L*_7_ from 0.8 mm to 1 mm. Only the first band will move down, while the remaining bands are fixed, as shown in Figure 13. 

**Figure 14:**
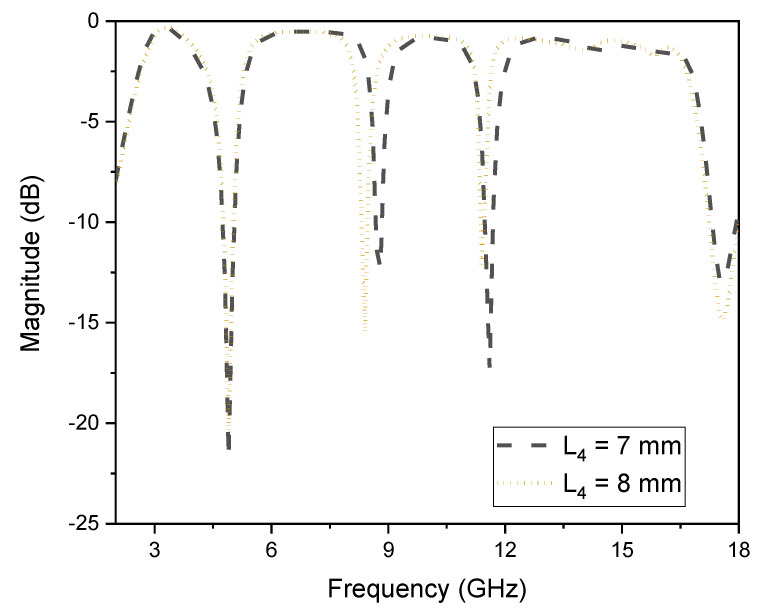
Validation of second band control w.r.t *L*_4_.

The second stopband at 8.3 GHz was realized due to the first odd mode of the resonator and by changing the length *L*_4_. Only the second band will change, while the first and third bands are constant, as shown in Figure 14. 

**Figure 15:**
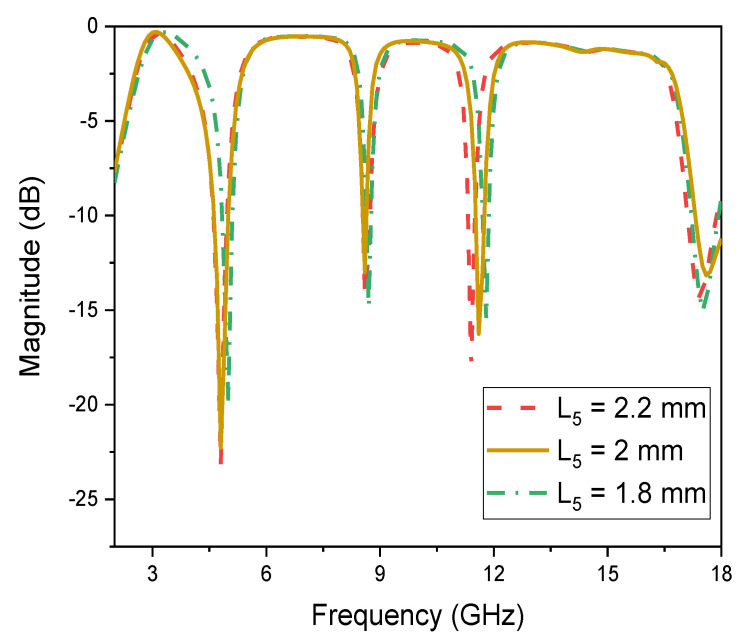
Validation of third band control w.r.t *L*_5_.

**Figure 16:**
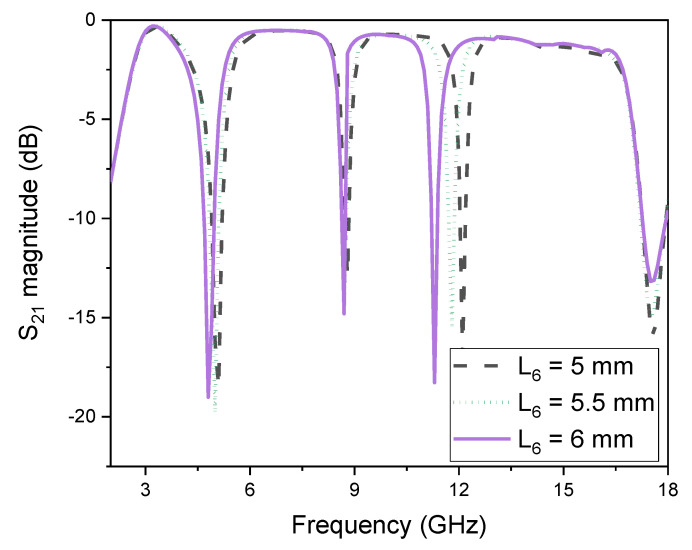
Validation of third band control w.r.t *L*_6_.

**Figure 17:**
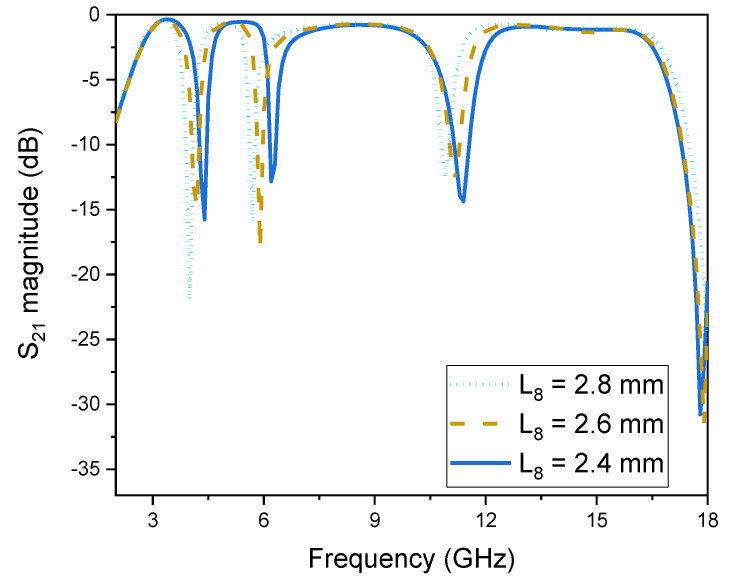
Validation of all bands w.r.t *L*_8_.

The third stopband at 11.5 GHz was realized due to the first even mode of the resonator, as derived in Equation (6). In Figure 15 and Figure 16, only the third band is changed widely by changing the lengths *L*_5_ and L_6_, while the other two bands almost remain constant. Moreover, all the stopbands are simultaneously decreased with the parameter *L*_8_ from 2.4 mm to 2.8 mm, as shown in Figure 17. This is because the parameter *L*_8_ is present in all the resonance equations, as derived above. 

The above results show that the presented filter has the capability to control all the stopbands separately. The corresponding insertion loss, bandwidth at the −10 dB attenuation level, and the rejection level of each stopband are listed in Table 3, while the theoretical, simulated, and measured results for each stopband are tabulated in Table 4, respectively. The slight deviations in the theoretical, experimental, and fabricated results are due to inevitable human errors in measurement, losses in SMA connectors and substrate material, and the effect of soldering.

**Figure 18:**
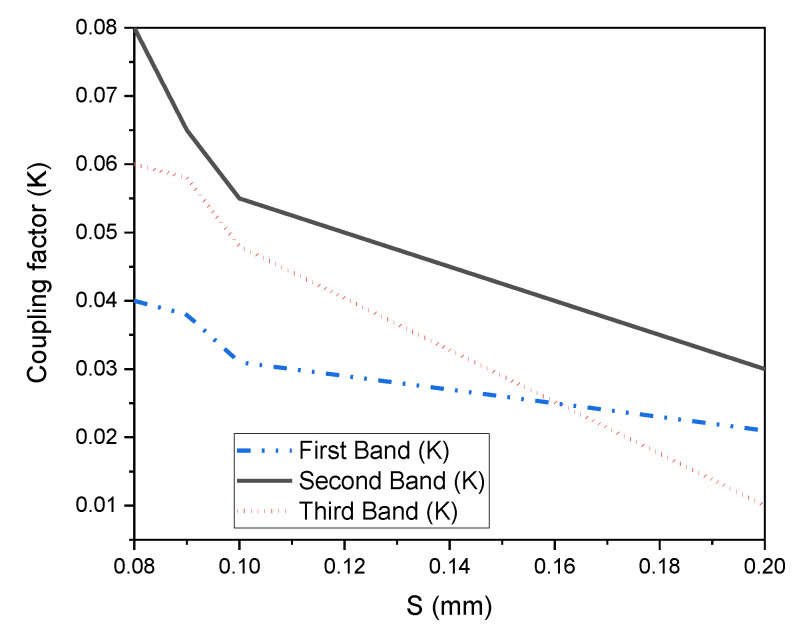
Coupling phenomena with respect to the parameter S.

Another important parameter is the coupling coefficient discussed by the authors [25,26]. As shown in Figure 12, the space S is responsible for the generation of coupling phenomena in the filter, and by increasing the space S from 0.08 mm to 0.2 mm, the coupling coefficient decreases, or vice versa, as shown in Figure 18, and is obtained due to Equation (21) by considering the values of Table 5.

(21) $$K_{e}=\frac{f_{2}{}^{2}-f_{1}{}^{2}}{f_{2}{}^{2}+f_{1}{}^{2}}$$

In above equation, *f*_2_ and *f*_1_ represents the upper and lower frequency of each stopband.

**Figure 19:**
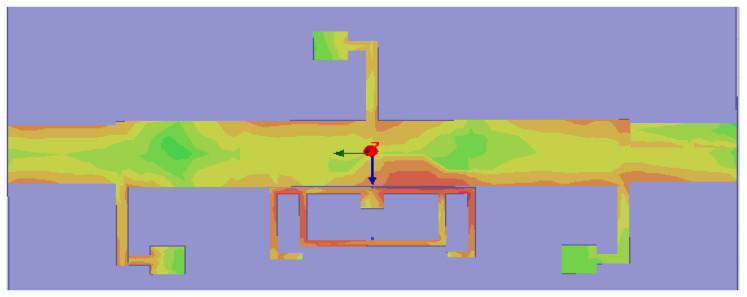
Current distribution graph of the triple-notched filter.

Figure 19 shows the simulated current distribution at the stopbands for validation of the resonance frequencies produced by the corresponding stubs. As discussed above, a novel T-shaped shorted stub-loaded resonator with a central square ring is utilized for the formation of notches in the ultra-passband response; therefore, most of the energy is absorbed by this resonator, as shown below.

Equations (22) and (23) described another important parameter in the design of UWB filters and antennas called the group delay (*τ*_*d*_) [27,28]. It is expressed as:

*τ*_*d*_ = *dφ*_21_(*ω*)/−*dω* (22)

*φ*_21_(*ω*) = arg *S*_21_(*ω*) (23)

**Figure 20:**
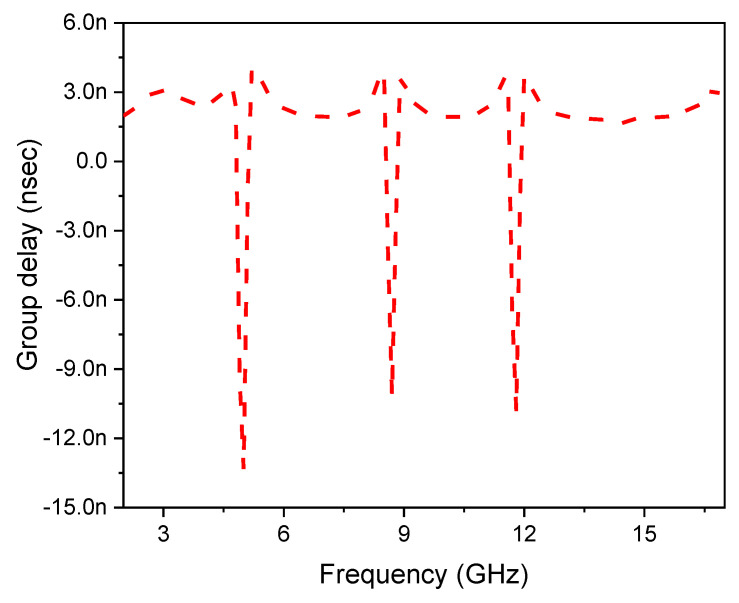
Group delay response of the proposed notched filter.

**Figure 21:**
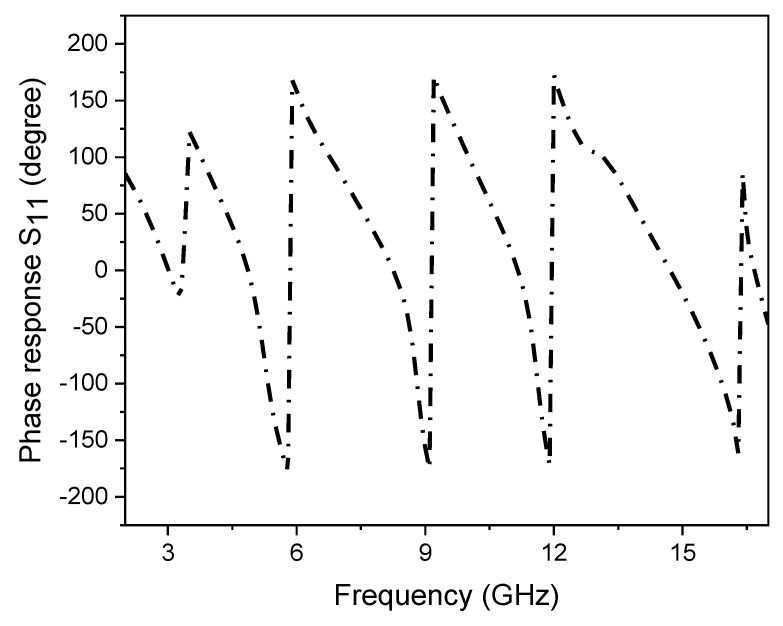
Phase response of the proposed triple-notched filter.

In above equations, *φ*_21_ and *τ*_*d*_ denote the parameter phase and group delay, respectively. As seen in Figure 20, the group delay is almost flat over the passband except at the notches, which guarantees that all the frequencies have the same group velocity and phase. This leads to a minimum frequency dispersion in the passband and a maximum at the stopbands [29,30]. The phase response of the proposed filter is illustrated in Figure 21.

**Figure 22:**
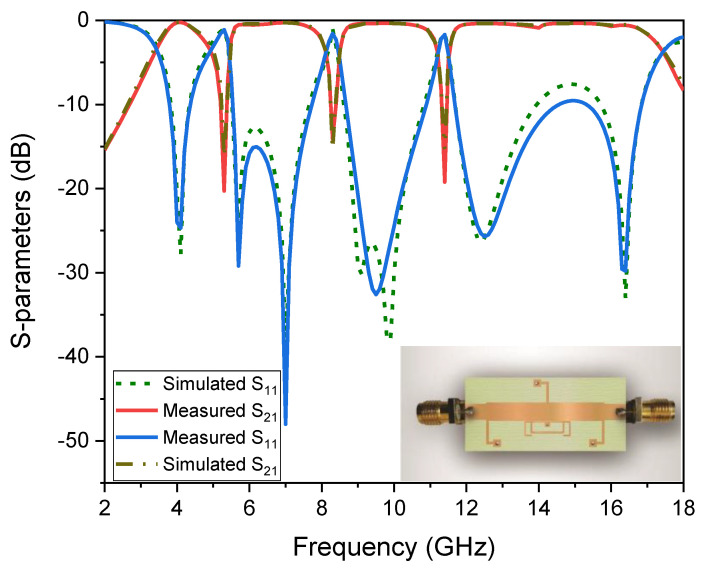
The measured and simulated results of the proposed triple-notched band filter.

The superiority of this research work is a simple topology to reduce the cost, which is then compared with the most recent published articles in reputed journals in terms of good FBW or wide absolute bandwidth, central frequency (CF), low IL at specific stopbands, high returns loss (RL), and independently controlled notch bands, which are listed in Table 6, respectively. Moreover, the final S_11_ and S_21_ frequency plots of the measured and simulated results with the fabricated prototype are shown in Figure 22. This verifies that the proposed architecture eliminates the potential interference in the ultra-wideband microwave applications and has the advantage of stopping and controlling the unwanted wireless bands according to the user’s needs.

## 6. Conclusions

In this article, a SUWB BPF with triple-notched bands was designed and fabricated using a UTL loaded with three folded λ/4 short-circuited stubs for the implementation of a wideband response, and then a quad-mode stub-loaded resonator was coupled to the basic SUWB-BPF for the rejection of unwanted signals at 4.9 GHz, 8.3 GHz, and 11.5 GHz with good return loss and sharp rejection, respectively. The circuit area covered by the proposed filter is 0.52 *λ_g_* × 0.32 *λ_g_*. A large fractional bandwidth of 141.1% with low insertion loss of less than 0.4 dB was achieved by loading the stubs at the appropriate distance on either side of the UTL. Due to its simple planar structure and excellent performance, the proposed filter can be integrated into future UWB wireless communication system for various applications. 

## Figures and Tables

**Table 1:** Dimensions in millimeter (mm) of the proposed SUWB-BPF with a stub-loaded resonator.

L	22.5	W	3.2	*L*_1_	4	*L*_2_	1
*L*_3_	1.5	*L*_4_	9	*L*_5_	2	*L*_6_	6
W_1_	1	*L*_8_	2.9	*L*_9_	1.2	*L*_7_	0.8
W_2_	0.3	W_3_	1.5	W_4_	0.5	S	0.1
R_1_	0.55	R_2_	0.3	PCB height	1.5	PCB	RO-4350
*ϵ_r_*	3.6	tan *δ*	0.0003	W_f_	3.1		

**Table 2:** Design table for finding the stopbands of the proposed filter.

Serial No.	Design Equation	Design Resonator Model	Theoretical Results	Simulated Results
1st stopband	$f_{even1}=\frac{(2n-1)c}{2(L_{2}+L_{3}+L_{4}+L_{5}+L_{6})\sqrt{\varepsilon_{eff}}}$	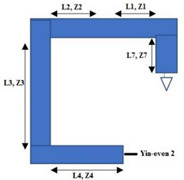	5.5 GHz	4.9 GHz
2nd stopband	$f_{odd}{}_{1}=\frac{c}{4(L_{1}+L_{2}+L_{3}+L_{4})\sqrt{\varepsilon_{eff}}}$	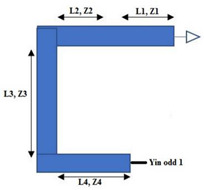	8.1 GHz	8.3 GHz
3rd stopband	$f_{odd2}=\frac{(2n+1)c}{4(L_{2}+L_{3}+L_{4}+L_{5}+L_{6})\sqrt{\varepsilon_{eff}}}$	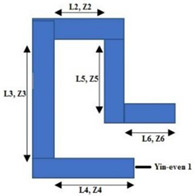	10.8 GHz	11.5 GHz

**Table 3:** Calculated properties of the stopbands.

Serial No.	Insertion Loss	Bandwidth (MHz)	Rejection Level
1ststopband	<0.5 dB	300	−20 dB
2ndstopband	<0.5 dB	290	−15 dB
3rdstopband	<0.5 dB	170	−19.3 dB

**Table 4:** Results variation of the stopbands.

Serial No.	Theoretical Results	Simulated Results	Measured Results
1ststopband	5.5 GHz	4.9 GHz	4.83 GHz
2ndstopband	8.1 GHz	8.3 GHz	8.51 GHz
3rdstopband	10.8 GHz	11.5 GHz	11.69 GHz

**Table 5:** Simulated result of “K” for different values of S.

S_1_ (mm)	1st Band (GHz)	2nd Band (GHz)	3rd Band (GHz)
0.08	0.08	0.06	0.04
0.09	0.065	0.058	0.038
0.1	0.055	0.048	0.031
0.2	0.03	0.01	0.021

**Table 6:** Performance comparison of various notched filters.

Ref. No.	Passband (GHz)	FBW (%)	IL/RL (dB)	C.F/BW (GHz)
[31]	3.3–10.7	105.7	0.9/15	4.4, 5.5, 7.6
[32]	3.1–10.6	117.0	1.25/>16	3.6, 5.2, 8.4
[33]	3.3–9.7	104	0.3/29	7.3
[34]	3.3–10.6	105	<0.5/14	8.95
[35]	3.1–11	112	0.66/35	6
[36]	3–10.9	110	0.9/>15	5.96, 8.15
[37]	3.58–10.07	95.1	<1.2/>15	5.53, 8.1
[38]	3.25–10.73	106	0.52/>19	5.6, 6.4, 8.03
This work	2.9–16.85	141.1	<0.4/>15	4.9, 8.3, 11.5

## Data Availability

Not applicable.
